# Line tension at lipid phase boundaries as driving force for HIV fusion peptide-mediated fusion

**DOI:** 10.1038/ncomms11401

**Published:** 2016-04-26

**Authors:** Sung-Tae Yang, Volker Kiessling, Lukas K. Tamm

**Affiliations:** 1 Center for Membrane and Cell Physiology, Department of Molecular Physiology and Biological Physics, University of Virginia, PO Box 800886, Charlottesville, Virginia 22908, USA

## Abstract

Lipids and proteins are organized in cellular membranes in clusters, often called ‘lipid rafts'. Although raft-constituent ordered lipid domains are thought to be energetically unfavourable for membrane fusion, rafts have long been implicated in many biological fusion processes. For the case of HIV gp41-mediated membrane fusion, this apparent contradiction can be resolved by recognizing that the interfaces between ordered and disordered lipid domains are the predominant sites of fusion. Here we show that line tension at lipid domain boundaries contributes significant energy to drive gp41-fusion peptide-mediated fusion. This energy, which depends on the hydrophobic mismatch between ordered and disordered lipid domains, may contribute tens of *k*_*B*_*T* to fusion, that is, it is comparable to the energy required to form a lipid stalk intermediate. Line-active compounds such as vitamin E lower line tension in inhomogeneous membranes, thereby inhibit membrane fusion, and thus may be useful natural viral entry inhibitors.

## Abstract (web summary)

HIV preferentially fuses with lipid membranes at the interface between ordered and disordered domains, but the mechanistic basis for this observation is not known. Here Yang *et al*. show that line tension at the lipid boundary contributes considerable energy to drive gp41 fusion peptide-mediated fusion.

## Graphical abstract

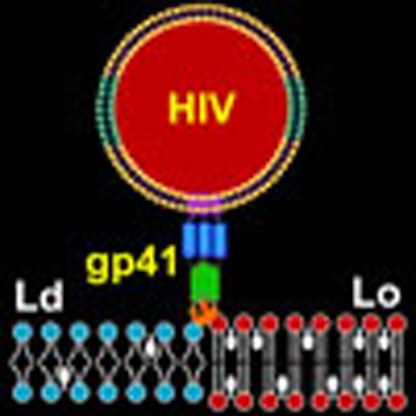

Human immunodeficiency virus (HIV) enters cells by membrane fusion at the plasma membrane^1^. After binding of the viral envelope glycoprotein gp120 to CD4 receptors and CXCR4 or CCR5 chemokine co-receptors on the plasma membrane of susceptible macrophages and T-lymphocytes, the viral envelope fusion protein gp41 undergoes a dramatic conformational change that ultimately leads to fusion of the two membranes^2^,^3^. The conformational change of gp41 exposes its N-terminal hydrophobic fusion peptide whose insertion into the target cell membrane is essential for fusion^4^,^5^.

The envelope of HIV and the plasma membrane of T-cells are both rich in cholesterol^6^,^7^,^8^. It is widely accepted that proteins and lipids are not randomly distributed in complex cell and derived viral membranes. Especially in the presence of high concentrations of cholesterol, certain lipids such as sphingolipids are thought to be organized into platforms, sometimes called lipid rafts, to carry out vital functions^9^,^10^. For example, lipid rafts are hypothesized to play important roles in signal transduction and intracellular trafficking^11^,^12^. Cholesterol and lipid rafts have also been implicated in T-cell activation^13^,^14^ and HIV entry into T-cells by membrane fusion^15^,^16^,^17^. In addition to fusion in viral entry, membrane fusion is a ubiquitous, fundamental biological process that has also been shown to be dependent on cholesterol in exocytosis of synaptic vesicles^18^, muscle development^19^ and fertilization^20^.

The requirement for lipid rafts, or more precisely liquid-ordered (Lo) lipid domains, for membrane fusion seems at first sight counter-intuitive. Fusion, which requires membrane bending and non-bilayer lipid intermediates^21^, is expected to be energetically unfavourable to occur in Lo regions of the membrane due to their rigid and tightly packed structures. However, we discovered recently that the edges rather than the central areas of Lo domains or lipid rafts are the sites of fusion of HIV pseudoviruses with model membranes containing micron-sized coexisting Lo- and liquid-disordered (Ld)-phase domains^22^. We also showed in that work that a model system using liposomes decorated with exposed gp41-fusion peptides behaved identically to the pseudoviruses with regard to fusion at Lo domain edges. However, the molecular mechanism and physical reasons why the fusion peptides of HIV pseudoviruses or related model systems prefer lipid phase boundaries rather than ordered or disordered bulk lipid phases are not yet understood. Lipid height mismatch, lipid distortions or membrane bending at the Lo–Ld phase boundaries could all contribute to the favourable energetics of fusion at phase boundaries^23^. To shed light on the mechanism of membrane fusion at lipid phase boundaries, we explore in the current work the effect of hydrophobic mismatch between Lo- and Ld-domain thickness, the effect of different sterols and the effect of a series of ‘linactants' on HIV fusion peptide (HIV-FP)-mediated membrane fusion (Fig. 1). Linactants are compounds that preferentially partition into lipid phase boundaries. They can be mixed chain lipids with hydrocarbon chains of different length or order, or non-lipid compounds as, for example, α-tocopherol or vitamin E. Most interestingly, vitamin E has been shown to suppress HIV-1 activation and has been suggested to mitigate the development of AIDS in the clinic^24^,^25^. Our studies show that line tension is the common denominator that determines the energetics of HIV gp41-fusion peptide insertion into the target membrane and subsequent membrane fusion. The work provides a first mechanistic molecular explanation why vitamin E might be beneficial in the clinic and also suggests developments for new strategies for therapies of AIDS. The mechanism we propose here for fusion and cell entry of HIV is likely general for many viral fusion proteins that are equipped with fusion peptides and perhaps even other cholesterol-dependent intracellular and developmental fusion systems.

## Results

### Fusion of complex model versus biological membranes

**Figure 1:**
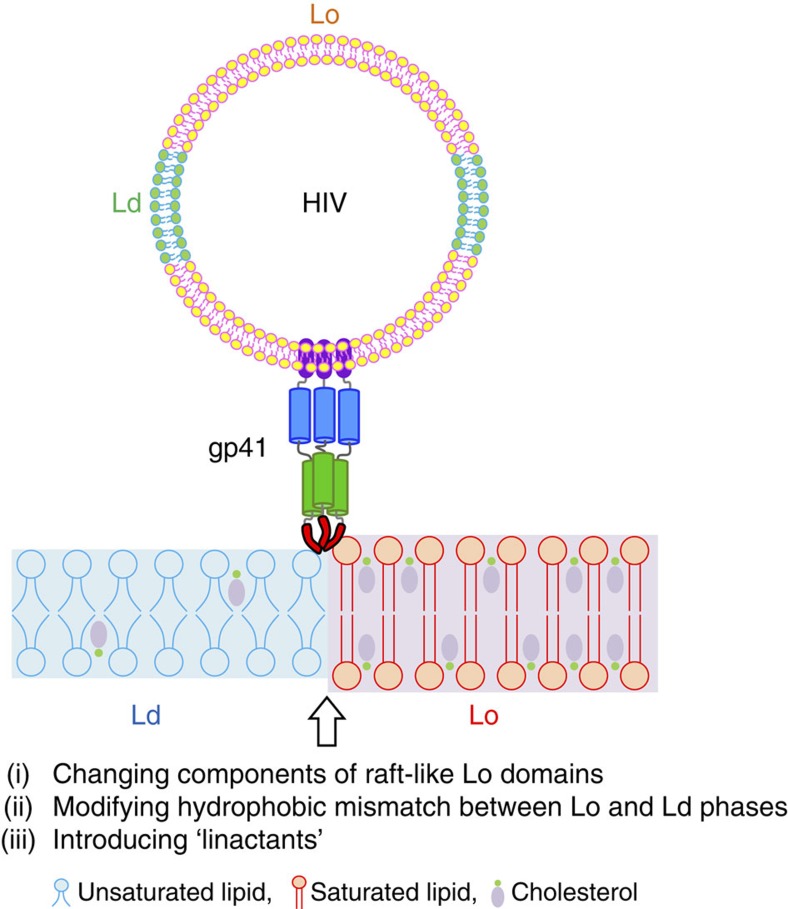
Experimental design to study effects of lipid phase boundaries on HIV gp41-mediated membrane fusion. Schematic representation of HIV gp41 interaction with Lo/Ld domain phase boundary. The fusion peptide of gp41 preferentially inserts and promotes membrane fusion at the interface between Lo and Ld phases. To address what molecular and physical properties are responsible for membrane fusion at these boundaries, we systematically modulated the interfaces by changing components of the Lo domains, modifying hydrophobic mismatch and introducing line-active molecules.

Rafts are difficult to observe in biological membranes due to their small size and dynamic nature, while raft-like domains have been characterized extensively in model membranes where lipid phase separation between Lo and Ld phases^26^,^27^ can be easily observed. Although we realize that the larger-scale model systems do not represent precise models for more transient membrane domains that are observed in cells, we believe that the model systems are still very informative for studying the prevailing physical interactions that must also apply to fusion peptide-membrane interactions in cells. The physical differences between adjacent membrane domains may be smaller in cell membranes than in the model systems, but on the other hand, they are more frequent and thus become a more dominant factor as the edge-to-surface area ratio increases with smaller domain sizes expected in cell compared with model membranes^23^,^28^,^29^. Therefore, we model lipid rafts in this study as ternary or quaternary lipid mixtures of sphingomyelin or saturated lipids, unsaturated lipids and cholesterol that form coexisting Lo and Ld domains that have been extensively studied by other authors^30^,^31^,^32^. Because of the higher chain order of sphingolipids and saturated phospholipids, lipid bilayers in cholesterol-rich Lo phases are thicker than those in cholesterol-poor Ld phases, leading to a height mismatch at the interfaces between coexisting phases (Fig. 1). Consequently, an interfacial energy called ‘line tension' arises at such phase boundaries. To systematically investigate the role of line tension on HIV-FP insertion and membrane fusion, we measured fusion in bulk systems and with single particles with hydrophobically mismatched coexisting Lo and Ld domains in large and giant unilamellar vesicles (LUVs and GUVs) and in supported lipid bilayers (SLBs) and correlate the fusion activities with observed interfacial lengths and line tensions. In addition, we observed the effects of several cholesterol analogues and linactants including vitamin E on Lo/Ld phase appearance and membrane fusion.

### Effect of raft- and non-raft-promoting components on fusion

**Figure 2:**
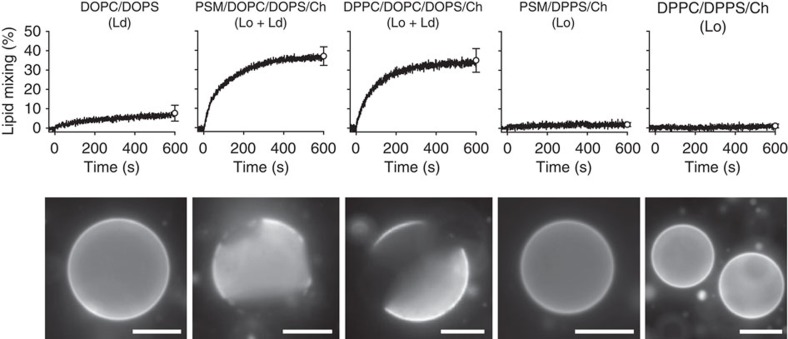
Comparison of the effect of Lo phase-promoting PSM and DPPC on lipid mixing mediated by HIV-FP. Lipid mixing with 1 μM HIV-FP added to 50 μM LUVs composed of from left to right DOPC/DOPS (3:1), PSM/DOPC/DOPS/Ch (2:1:1:1), DPPC/DOPC/DOPS/Ch (2:1:1:1), PSM/DPPS/Ch (2:1:1) and DPPC/DPPS/Ch (2:1:1; top row). Fluorescence micrographs of GUVs with corresponding lipid compositions and labelled with 0.1 mol% Rh-PE (bottom row). Scale bars, 10 μm. Error bars are s.d. of three replicates.

**Figure 3:**
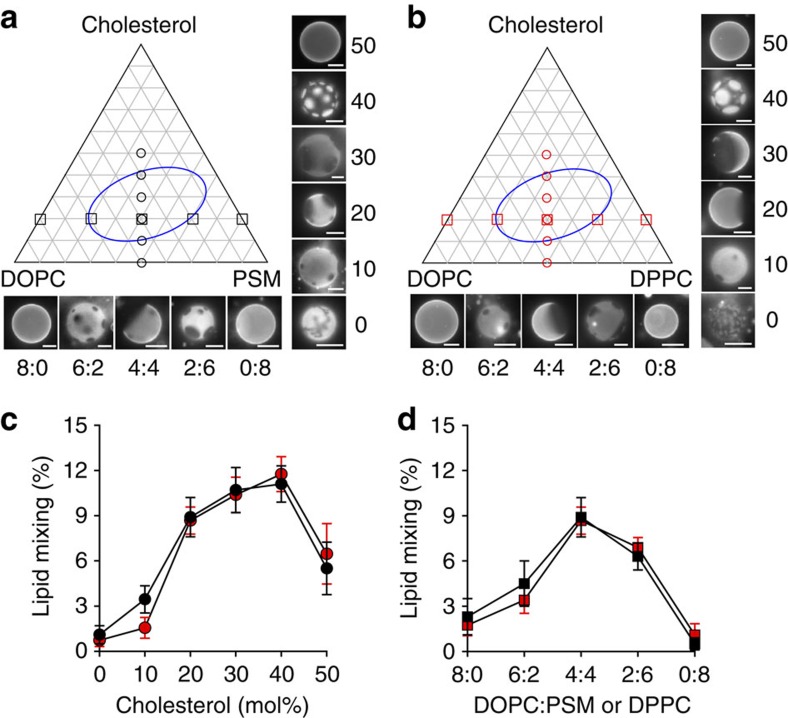
Lipid phase-dependent membrane fusion mediated by HIV-FP. Ternary lipid mixtures composed of (**a**) PSM/DOPC/Ch or (**b**) DPPC/DOPC/Ch with variable ratios were prepared as indicated in the triangular phase diagrams. Fluorescence micrographs of GUVs at constant 1:1 saturated:unsaturated lipid ratio with increasing cholesterol concentrations (0, 10, 20, 30, 40 and 50 mol% on perpendicular axes of the phase diagrams) and GUVs at constant 20 mol% cholesterol with variable DOPC/PSM (or DPPC) ratios (on horizontal axes of the phase diagrams). Scale bars, 10 μm. (**c**) Cholesterol- or (**d**) DOPC/PSM (or DPPC) ratio-dependent lipid mixing of 100 μM LUVs induced by 5 μM HIV-FP. LUVs are composed of the same lipid mixtures as used in **a**,**b**. PSM/DOPC/Ch and DPPC/DOPC/Ch data are shown with black and red symbols, respectively. Data are mean±s.d. from three experiments.

First, we examined which components of typical raft lipid mixtures promote or do not promote fusion mediated by the HIV-FP. Ld phase LUVs composed of dioleoyl-phosphatidylcholine (DOPC) and dioleoyl-phosphatidylserine (DOPS) show limited lipid mixing and corresponding GUVs are uniformly stained with rhodamine-dioleoyl-phosphatidylethanolamine (Rh-PE; Fig. 2, left panels). When raft-forming palmitoyl-sphingomyelin (PSM) or dipalmitoyl-phosphatidylcholine (DPPC) and cholesterol are mixed in, Lo/Ld phase separation occurs in GUVs and HIV-FP-mediated lipid mixing is dramatically enhanced (Fig. 2, second and third panels). When the Ld-forming components DOPC and DOPS are replaced with dipalmitoyl-phosphatidylserine (DPPS) in these mixtures, uniformly stained Lo phase GUVs are produced, and the corresponding LUVs are not fusogenic (Fig. 2, last two panels). The fusion efficiencies and rates with two-phase liposomes are not significantly different when PSM or DPPC are used as the saturated lipid components. Hence the amide versus ester linkage of the *sn*-1 acyl chain has no effect on fusion. Qualitatively very similar results are observed in fusion experiments with ternary lipid mixtures of PSM/DOPC/Ch or DPPC/DOPC/Ch, that is, when the acidic components DOPS or DPPS in the mixtures were replaced with DOPC and DPPC, respectively (Fig. 3). Figure 3a,b show ternary phase diagrams with images of GUVs of indicated compositions and Fig. 3c,d show the corresponding fusion data. There is a strict correlation between Lo/Ld phase separation and HIV-FP-mediated fusion activity. The only difference between the data with and without phosphatidylserine is that the fusion activities are quantitatively lower in the absence of acidic lipids, which has been previously observed and explained by the positive charges on the fusion peptide^22^.

The reason that Lo/Ld phase-separated membranes are more fusogenic than pure Lo or Ld single-phase membranes is that HIV-FP LUVs preferentially bind to Lo/Ld phase boundaries. This can be shown directly by two-colour total internal reflection fluorescence (TIRF) microscopy using phase-separated SLBs (Supplementary Fig. 1)^22^. Using a previously developed assay^33^ we quantified the binding of LUVs to three different regions (Lo, Ld or boundary) of the two-phase bilayer. About 60% of all HIV liposomes bound in the Lo/Ld interface regions, ∼32% in the Lo regions and ∼8% in the Ld regions, irrespective of whether the rafts were formed with PSM or DPPC as the saturated lipid component (Supplementary Fig. 1). Similar results were observed when HIV-FP LUV liposomes were bound to phase-separated GUVs (Supplementary Movie 1).

**Figure 4:**
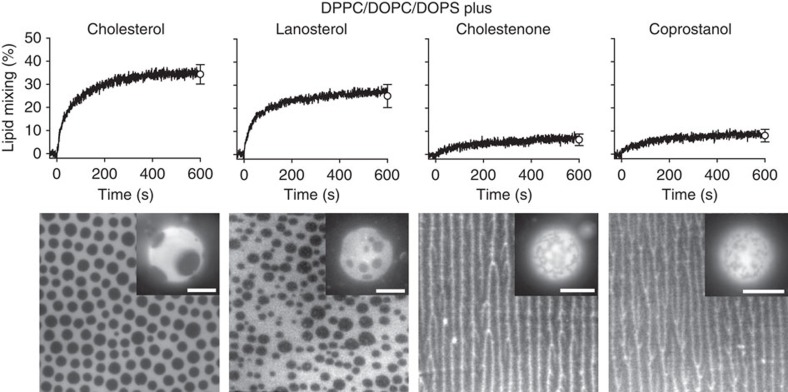
Effect of different sterols on Lo domain formation and membrane fusion. Lipid mixing with 1 μM HIV-FP added to 50 μM LUVs composed of DPPC/DOPC/DOPS/sterol (2:1:1:1; top row). Sterols from left to right are cholesterol, lanosterol, cholestenone and coprostanol as indicated. Fluorescence micrographs of supported lipid monolayers and GUVs (insets) with corresponding lipid compositions and labelled with 0.1 mol% Rh-PE (bottom row). The scale of all lipid monolayers is 60 × 60 μm^2^ and scale bars in insets are 10 μm. Error bars are s.d. of three replicates.

Cholesterol and lanosterol are known to be promoters of Lo domain formation, while cholestenone and coprostanol are known to inhibit Lo domain formation^34^. In agreement with previous studies^35^, cholesterol and lanosterol induced round Lo domains on lipid monolayers and GUVs, whereas lipid mixtures containing cholestenone and coprostanol lead to irregular domain structures in otherwise identical quaternary lipid mixtures with phosphatidylserine (Fig. 4). The patterns observed with cholestenone and coprostanol consist of an intricate network of fiber-like features in SLBs and irregular domains in GUVs, which is characteristic of some coexisting gel- and fluid-phases^36^. LUVs containing the two Lo promoting sterols show efficient lipid mixing in the presence of HIV-FP, while LUVs containing the two Lo inhibiting sterols did not fuse efficiently (Fig. 4). These experiments prove that it is not the detailed chemical structure of these sterols, but rather their ability to form coexisting Lo and Ld domains that determines their fusogeneity in the presence of the HIV-FP.

### Effect of Lo/Ld phase hydrophobic mismatch on fusion

**Figure 5:**
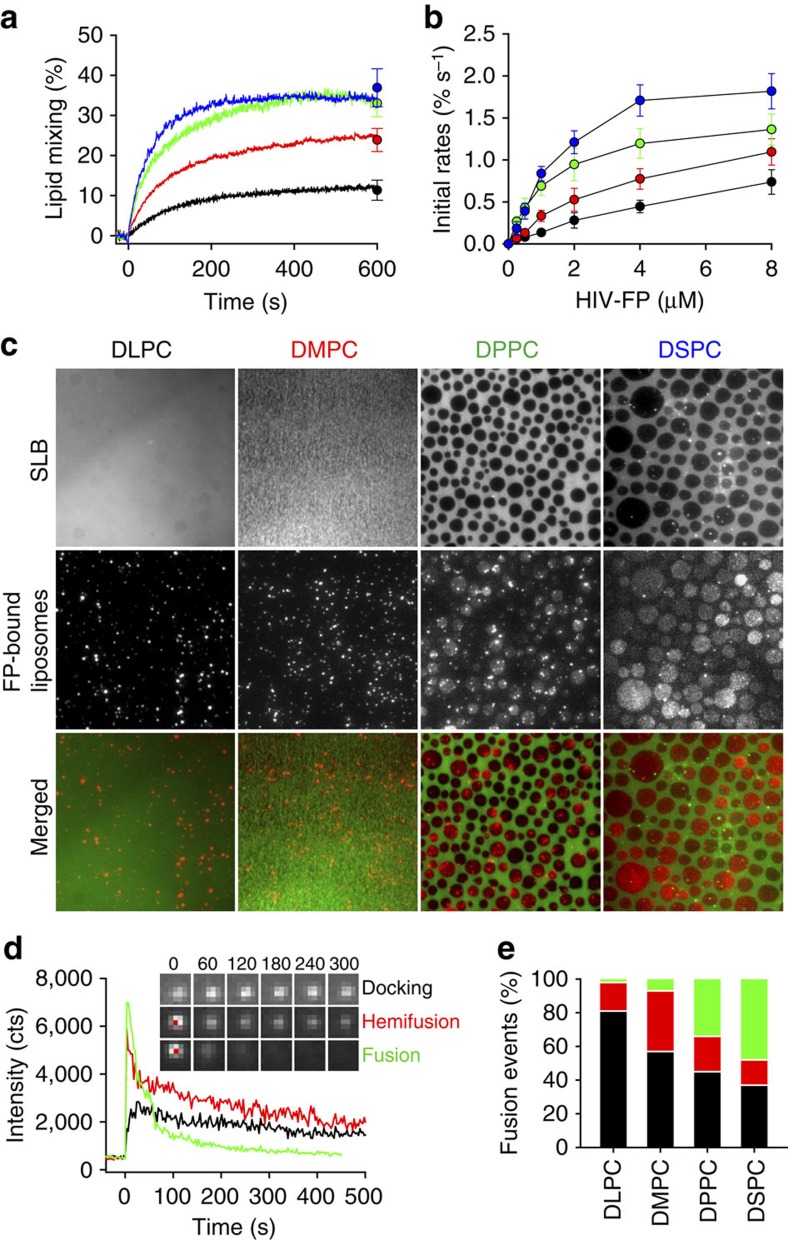
Effect of hydrophobic mismatch on Lo domain formation and membrane fusion. (**a**) Effect of saturated lipid component on lipid mixing. 1 μM HIV-FP was added to 50 μM LUVs composed of DSPC/DOPC/DOPS/Ch (2:1:1:1; blue), DPPC/DOPC/DOPS/Ch (2:1:1:1; green), DMPC/DOPC/DOPS/Ch (2:1:1:1; red) and DLPC/DOPC/DOPS/Ch (2:1:1:1; black). (**b**) Initial rates of lipid mixing as function of HIV-FP concentration. Same colour designations are used as in **a**. Data are mean±s.d. from three experiments. (**c**) Fusion between LUVs and SLB. LUVs and SLBs were composed of saturated lipid/DOPC/DOPS/Ch (2:1:1:1). The saturated lipids are DLPC, DMPC, DPPC or DSPC as indicated. LUVs were added to SLBs which were pre-incubated with 5 μM HIV-FP for 10 min. The images were acquired 20 min after vesicle addition. Fluorescence micrographs of SLBs labelled with 0.1 mol% Rh-PE (top row), TIRF micrographs of bound/fused LUVs labelled with 0.5 mol% DiD on SLB (middle row), and merged images (bottom row). The scale of all images is 64 × 64 μm^2^. (**d**) Representative single-LUV fusion events on SLBs including docking, hemifusion and full fusion. Time zero is defined as the first frame with a visible liposome. The insets show TIRF microscopy images of representative times (s) for each type of fusion event. The scale of all inset images is 2.5 × 2.5 μm^2^. (**e**) Relative frequencies of single-LUV docking (black), hemifusion (red) and full fusion (green) events.

We next examined if membrane fusion is related to the height mismatch at the interface between Lo and Ld phases. To systematically modulate the thickness difference at the domain boundaries, we used saturated lipids with various acyl chain lengths (dilauroyl-phosphatidylcholine (DLPC), dimyristoyl-phosphatidylcholine (DMPC), DPPC and distearoyl-phosphatidylcholine (DSPC)) while keeping the unsaturated lipids DOPC and DOPS, and cholesterol in the mixtures. The widths of these bilayers have been measured by x-ray diffraction and other methods^37^. The widths of neat fluid bilayers increase by ∼4 Å for each two methylene segments added to both hydrocarbon chains of the saturated lipids and the thickness of DOPC bilayers is intermediate between those of fluid phase DMPC and DPPC. Typical fluorescence images of lipid monolayers and GUVs are shown in Supplementary Fig. 2. We observed phase separation in the mixtures containing DPPC or DSPC, whereas only single phases were observed with DLPC or DMPC. The domain sizes and shapes in the supported and non-supported membranes initially look similar. However, because the domains in GUVs tend to merge and eventually form only one or a few large domains, the supported membranes revealed additional details. The size of the Lo domains in mixtures containing DSPC was larger than those in mixtures containing DPPC. This indicates that the presence of different line tensions are defining the boundaries between Lo and Ld phase regions in these lipid mixtures, although kinetic factors could also play a role in determining the observed domain sizes. Although domains cannot be seen in the DLPC-containing lipid bilayers and in DMPC-containing lipid bilayers in GUVs at the resolution of standard light microscopy, extrapolation from the other images suggests that optically unresolved nanoscopic domains may be present in these bilayers. It is well known that lipid bilayers increase their fluid phase thickness as more cholesterol is incorporated^38^. In phase-separated systems more cholesterol is expected to partition into the saturated lipid phase than into the unsaturated lipid phase, thereby increasing the thickness of the DLPC, DMPC, DPPC and DSPC-rich regions more (on the order of 15% at 30–40 mol% cholesterol in that phase) than that of the DOPC-rich regions (almost not affected). Therefore, the DLPC/cholesterol Lo phase becomes almost equally thick as the DOPC/cholesterol Ld phase and the mismatch increases as the hydrocarbon chains increase to DMPC, DPPC and DSPC in the phase-separated bilayers. (If <30 mol% cholesterol were incorporated into the Lo phase, then the DLPC/cholesterol phase would still be thinner than the DOPC/cholesterol phase, which would match the DMPC/cholesterol phase more closely.) To test if the hydrophobic mismatch between the Lo and Ld domains affect membrane fusion, these same lipid mixtures were used in lipid mixing experiments of LUVs (Fig. 5a,b). HIV-FP induced the most rapid and efficient fusion of DSPC-containing liposomes, decreased gradually with liposomes containing DPPC and DMPC, and was lowest with DLPC-containing liposomes. Importantly, the lipid mixing efficiency increased linearly with the acyl chain length of the saturated lipids used, indicating that it depends linearly on the hydrophobic mismatch at the Lo/Ld interfaces, assuming that the Lo phase contains >30 mol% cholesterol.

Next, we investigated by TIRF microscopy HIV-FP-mediated docking and fusion of single liposomes to SLBs. The top panels in Fig. 5c show fluorescent micrographs of SLBs prepared with the same four lipid compositions used in the lipid mixing experiments above. LUVs were added to their corresponding SLBs, which were pre-incubated with HIV-FP. After 20 min, bright spots corresponding to unfused liposomes were observed in DLPC- or DMPC-containing SLBs, whereas relatively bright round domains appeared at the boundaries and within Lo phase domains in DPPC- or DSPC-containing SLBs, indicating that LUVs fused with these SLBs (Fig. 5c, middle panels). Supplementary Movie 2 shows the time course of docking and fusion of individual liposomes. To analyse these fusion events, peak fluorescence intensities from each bound liposome were extracted as function of time. Three types of events can be distinguished: (1) docking only, identified by a constant fluorescence intensity over time, (2) docking followed by hemifusion, identified by a decay of the fluorescence to around one half of the original peak intensity and (3) docking followed by full fusion, identified by a complete decay of the fluorescence intensity (Fig. 5d). Several hundred liposomes (313 with DLPC, 328 with DMPC, 421 with DPPC and 377 with DSPC) were analysed under each condition and the relative frequencies of the different types of events are shown in Fig. 5e. Total fusion efficiencies, that is, the proportion of docked liposomes that fused, were 3, 7, 34 and 48% for the membranes containing DLPC, DMPC, DPPC and DSPC, respectively. This again indicates a strong dependence of the fusion efficiency on the hydrophobic mismatch between the Lo and Ld domains in these systems. In addition, fusion observed with model membranes containing the shorter saturated acyl chain lipids was more likely arrested at a hemifusion state that did not proceed to full fusion. In contrast, events that proceeded all the way to full fusion were much more often observed with phase-separated Lo/Ld liposomes that contained the longer saturated acyl chains (Fig. 5e).

### Effect of linactants on membrane fusion

**Figure 6:**
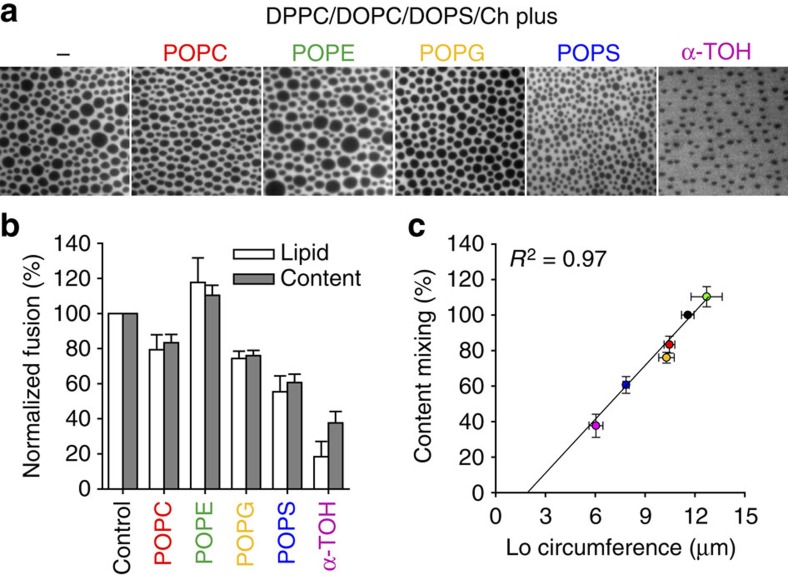
Effect of linactants on Lo domain formation and membrane fusion. (**a**) Fluorescence micrographs of supported lipid monolayers composed of DPPC/DOPC/DOPS/Ch (2:1:1:1) (control) with 20 mol% linactants (hybrid phospholipids and α-TOH) as indicated. The statistics of several observed parameters on the Lo domains as calculated by ImageJ are shown in Supplementary Fig. 3a. The scale of all images is 40 × 40 μm^2^. (**b**) Effect of linactants on membrane fusion mediated by HIV-FP. The extent of lipid (white bars) and content (grey bars) mixing was measured 10 min after addition of 1 μM HIV-FP to 50 μM LUVs composed of the same lipid mixtures as in **a**. (**c**) The dependence of content mixing on the circumference of Lo domains shows a linear relationship (correlation coefficient *R*^2^=0.97). The coloured data points correspond to the colours of the added linactants in **a**,**b**. The direct correlation of content (or lipid) mixing with the circumference of the domains indicates a critical role of the Lo/Ld interface line tension in membrane fusion. Data are mean±s.e.m. from three experiments.

Hybrid lipids bearing a saturated and an unsaturated chain and α-tocopherol (α-TOH or vitamin E) can act as linactants in mixed phase lipid bilayers^39^,^40^. Hybrid lipids have been proposed to decrease the line tension at domain boundaries by adsorbing to the interface between Lo and Ld phases. We investigated four hybrid lipids (POPC, POPE, POPG and POPS; PO stands for palmitoyl-oleoyl- in these phospholipids with choline, ethanolamine, glycerol and serine headgroups, respectively) and α-TOH on their effects on line tension and on HIV-FP-mediated membrane fusion. We added 20 mol% hybrid lipids or α-TOH to DPPC/DOPC/DOPS/cholesterol (2:1:1:1) supported monolayers and observed the respective phase behaviours. Example fluorescent micrographs are shown in Fig. 6a and a quantitative analysis of the observed areas, radii, circumferences and fractions of Lo phase domains in these membranes are summarized in Supplementary Fig. 3a. Incorporation of the hybrid lipids decreases the Lo domain sizes in the following order: POPE<POPC<POPG<POPS, with POPE having no and POPS having the strongest effect on domain size reduction (and perhaps some kinetic factors), suggesting that these hybrid lipids alter the line tension between different membrane phase regions to different degrees. Incorporation of α-TOH substantially decreased the average size of the Lo domains. The effects of hybrid lipids and α-TOH on HIV-FP-induced fusion measured by lipid and content mixing are shown in Fig. 6b. Incorporation of POPE caused a slight increase in the fusion efficiency, while incorporation of POPC, POPG or POPS reduced the amount of membrane fusion. Comparing the fusion efficiency with number, area, radius and fraction of the Lo domains (Supplementary Fig. 3b,c) shows that the fusion efficiency correlates extremely well (*R*^2^=0.97) with the circumference of the Lo domains (Fig. 6c), as expected if a property such as tension of that line is driving fusion in these systems. We further investigated the effect of POPC (Supplementary Fig. 4) and α-TOH (Supplementary Fig. 5) on the Lo domain behaviour and lipid mixing efficiencies in two types of ternary lipid mixtures composed of DPPC/DOPC/Ch (2:2:1) and PSM/DOPC/Ch (2:2:1). Increasing the POPC content affected the Lo phase domain structures in DPPC/DOPC/Ch bilayers, but not in PSM/DOPC/Ch bilayers. Increasing the POPC content also decreased the fusion efficiency of DPPC/DOPC/Ch liposomes, but did not change them with PSM/DOPC/Ch liposomes (Supplementary Fig. 4). Although the fusion activities still correlate with Lo domain appearances in both cases, the reason why the linactant POPC has a different effect on PSM than on DPPC Lo domains is presently not known and beyond the scope of the current study. Adding increasing amounts of α-TOH to the above lipid mixtures decreased the size of Lo domains and suppressed lipid mixing in both systems, supporting the notion that α-TOH inhibits fusion by its linactant activity on inhomogeneous lipid membranes.

## Discussion

A central conclusion of this work is that line tension, which defines the existence and size of ordered lipid domains in model and biological membranes, is the main reason why the edges of membrane domains are the preferred sites for HIV entry by gp41-mediated membrane fusion. Experimentally modifying line tension by changing the lipid components of ordered and disordered lipid domains, systematically varying the hydrophobic mismatch between the thicker Lo and thinner Ld regions of the membrane, and adding linactants to decrease the line tension at Lo/Ld interfaces all lead to the conclusion that line tension is indeed the common denominator that explains all observed effects. We have thus discovered a new molecular mechanism that contributes to HIV gp41-fusion peptide-mediated membrane fusion and perhaps other membrane fusion events in complex heterogeneous membranes.

**Figure 7:**
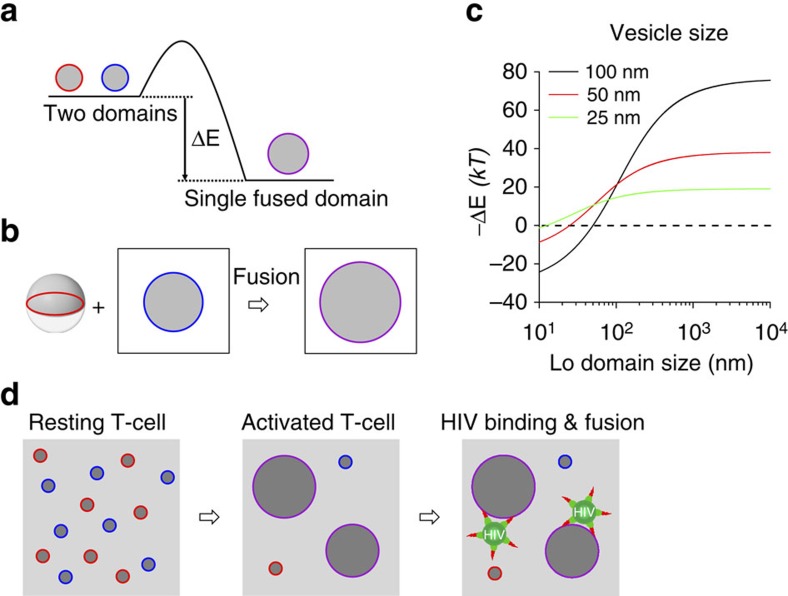
Release of boundary energy by domain coalescence. (**a**) Schematic diagram illustrating the change of boundary energy by domain coalescence. (**b**) A symmetric vesicle with two equally sized Lo- and Ld-phase hemispheres fuses with a Lo domain in a planar membrane. (**c**) Change of boundary energy for the reaction shown in **b** as a function of Lo domain size in the planar membrane for vesicles of different sizes. Domain fluctuations are not considered in this simple geometrical model (see text). (**d**) Schematic diagram illustrating biological implications of domain coalescence in T-cell activation on HIV binding and fusion at domain boundaries. The small domains shown in resting T-cells may actually be dynamic fluctuating clusters or nanodomains of receptors and lipids^29^, but even then, the concept of lateral assembly of multiple clusters and domain growth during T-cell activation is still valid.

There is a rich literature describing line tension in heterogeneous lipid membranes^26^,^27^,^32^,^41^,^42^,^43^,^44^,^45^. In a most simplistic model, which ignores domain fluctuations, the boundary energy of an isolated Lo domain in a Ld membrane is given by *E*=*γL*, where *γ* is the line tension and *L* is the circumference of the domain^44^. Since the perimeter/area ratio is smaller for large than for small domains, energy can be gained by merging small into large domains (Fig. 7a). We propose that this effect could provide a driving force for fusing a phase-separated vesicle or a cholesterol-rich HIV particle with domains in model or cellular target membranes of heterogeneous lipid composition. In this oversimplified model, we estimate the magnitude of this effect by calculating the related energy gain, defined as –ΔE, that is, the negative reduction in free energy, on fusion of a two-domain vesicle consisting of a single-Lo and a single-Ld hemisphere with a single-Lo domain in a planar target membrane (Fig. 7b). Assuming that the line tension *γ* is 1 pN (ref. 44), we find that the energy gain from line tension reduction by simple geometric transformation on fusion of 25, 50 and 100 nm vesicles increases sigmoidally as a function of the log of the size of the domain in the target membrane and becomes positive (free energy reduced), that is, favourable for fusion, only when that domain reaches half the size of the vesicle (Fig. 7c). The reason why fusion between large vesicles and small domains in a planar membrane is unfavourable in this oversimplified system is that the ‘two pole-cap' three-dimensional vesicle needs to integrate into the flat membrane and transform into a planar geometry with the same surface area, which leads to a longer and not shorter Lo/Ld interface under these extreme conditions. In a more realistic situation, a vesicle might have multiple domains with sizes more comparable to those in the planar membrane, which would make fusion energetically favourable even for large vesicles. The magnitude of the effect of line tension on fusion is on the order of several tens of *k*_*B*_*T*, depending on vesicle and domain size, but could of course be smaller or larger if more complex lipid systems with smaller line tensions or the merger of multiple domains would be considered in more realistic situations. Still, the effect of energy gain due to a change in line tension on fusion is similar in magnitude to the energy barrier that has to be overcome for stalk formation and fusion pore opening in membrane fusion and that is also commonly assumed to be on the order of several tens of *k*_*B*_*T* (ref. 21). Therefore, the afore-discussed considerations of change in line tension deserve attention as a driving force for fusion, especially in situations when the target membrane contains domains that are at least as large as the vesicles or viruses. It is very interesting to note in this context that T-cell activation is well known to lead to the clustering of cholesterol-rich membrane domains and that this effect has been shown to precede HIV entry into the cell^13^,^14^. Although receptor and co-receptor clustering may also be responsible for these observed biological effects, our new results suggest a new additional molecular mechanism that could drive fusion of HIV particles with activated T cells and thereby likely contributes to HIV entry (Fig. 7d).

**Figure 8:**
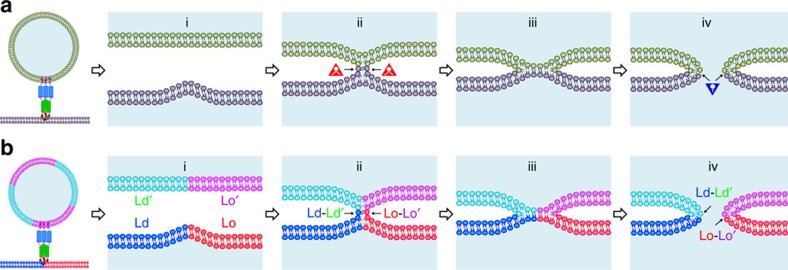
Extension of stalk-pore model of membrane fusion to heterogeneous lipid bilayers containing lipid rafts. (**a**) Schematic diagrams illustrating different steps of membrane fusion in the standard stalk-pore model with homogeneous membranes: (i) close contact of two lipid bilayers with point-like protrusion, (ii) lipid stalk connecting two bilayers, (iii) hemifusion diaphragm and (iv) fusion pore. Lipids with negative spontaneous curvature (red triangles) stabilize the stalk and lipids with positive spontaneous curvature (blue inverted triangles) stabilize the fusion pore. (**b**) Stalk-pore model extended to fusion of lipid bilayers with coexisting Lo/Ld domains. The domain boundaries generate additional energy for membrane fusion by reduction of line tension energy. These boundaries induce local curvature and defects that facilitate fusion peptide insertion at different steps in the extended stalk-pore model (see text, for more detail).

The stalk-pore model has been widely accepted as a common mechanism for membrane fusion^46^,^47^,^48^. Figure 8a illustrates progression through a few intermediate states according to this model. These intermediates include (i) close contact of the two membranes, (ii) a lipid stalk, (iii) a hemifusion diaphragm and (iv) an initial fusion pore. Proteins catalyse this process and it is still debated how exactly they may be integrated structurally and mechanistically into this model. Regardless, one of the current challenges is to explain how intermediate structures of fusion proteins lower the energy barriers for transitions between the different fusion intermediates. Since the lipid bilayers of both membranes need to be dramatically rearranged during fusion, the types, shapes and other physical properties of the participating lipids are also believed to be critically involved in the fusion process. For example, cone-shaped lipids that promote negative spontaneous curvature are believed to stabilize lipid stalks, while positive spontaneous curvature-promoting lipids are thought to stabilize early fusion pores (Fig. 8a).

Even though biological membranes are clearly not homogeneous, membrane heterogeneity has so far received only little attention in discussions of mechanisms of membrane fusion. This study begins to fill this gap and provides new insights into how the stalk-pore model may be adapted for the case of heterogeneous membranes, in which the reduction of line tension and fusion peptide insertion into the Lo/Ld interface provide new energetic drivers of heterogeneous membrane fusion. The following modifications to the classical stalk-pore model should be considered with heterogeneous membranes (Fig. 8b): (i) It is generally easier to bend membranes at fault lines between Lo and Ld phases. This will facilitate the close contact step. Fusion peptide insertion is also favoured at these lines gaining more energy than inserting the peptide in homogeneous membranes^22^,^49^. (ii) Since it is energetically favourable to align phase boundaries in stacked lipid bilayers^50^, stalks may be favoured to form at sites between such aligned boundaries in both membranes. Evidence that phase boundaries in not just one, but both participating membranes are beneficial for fusion has been presented before in a model system^22^, and it is reasonable to assume that similar physical principles also apply to biological membranes. (iii) A hemifusion diaphragm may form or may be bypassed as an intermediate. (iv) Fusion pore formation requires the creation of positive membrane curvature. Since phase boundaries create positive curvature^26^, pore formation should be facilitated in regions of phase boundaries. The overall energy gain from the reduction in line tension on fusion at membrane phase boundaries provides additional energy to drive fusion at these sites.

The effect of membrane heterogeneity and phase separation on membrane fission, which is also a very common and ubiquitous cell biological process, has received more attention than its effect on membrane fusion. Experimental and theoretical work showed that Lo/Ld phase coexistence facilitates fission of model membranes and that line tension at the phase boundaries provides a driving force for membrane fission^26^,^41^,^42^. The common findings of these previous studies on fission and our studies on fusion reported here are not surprising because membrane fusion can be considered as the reverse process of membrane fission. Indeed, membrane fusion shares many mechanistic properties with membrane fission^43^. Membrane microdomains may be formed by protein–protein, lipid–lipid or protein–lipid interactions and all of these interactions may produce local curvature and line tension at membrane domain interfaces that can provide energy for protein-mediated membrane fission or fusion. Line tension and local curvature thus play key roles in the regulation of both processes.

A very interesting result of the current work is that α-TOH or vitamin E reduces line tension and thus HIV-FP-mediated membrane fusion. Low levels of α-TOH are often found in HIV-infected individuals and it has been proposed that higher levels may decrease the risk of infection with HIV and the emergence of HIV resistance in the population^24^,^25^. Our finding that α-TOH inhibits HIV-FP-mediated membrane fusion by acting as a linactant provides a plausible explanation for the inhibitory effect of α-TOH on HIV infection. Since vitamin E is a natural substance, it should not have very serious side-effects when given to patients in doses that partially protect them from infection. It will be interesting to see if this general concept of linactant therapeutics can be extended to similar compounds that could be developed into future drugs.

In summary, we found that line tension at heterogeneous membrane domain boundaries is an important factor that enhances HIV gp41-fusion peptide-mediated membrane fusion. Simple geometric considerations show that line tension will be reduced after fusion. Additionally, fusion proteins insert more easily and membranes are prone to bend more readily at the fault lines of membrane domain boundaries. Although shown here for Lo/Ld domain boundaries that are created by lipid–lipid interactions, the same principles likely apply also to domain boundaries that are created by protein–lipid or protein–protein interactions in more complex membranes. The symmetry between membrane fusion and membrane fission dictates that very similar principles should also apply to membrane fission. Since membrane fusion and fission are highly ubiquitous processes in cell biology, we believe that the results of this work likely apply to many other membrane fusion and fission systems that are essential for the well being and pathology of eukaryotic cells.

## Methods

### Materials

All lipids were from Avanti Polar Lipids (Alabaster, AL). 1,1′-dioctadecyl-3,3,3′,3′-tetramethylindodicarbocyanine perchlorate (DiD), aminonaphthalene-1,3,6-trisulfonic acid (ANTS) and p-xylene-bis-pyridinium bromide (DPX) were from Molecular Probes (Invitrogen, Carlsbad, CA). Cholesterol, lanosterol, cholestenone and coprostanol were purchased from Sigma (St Louis, MO). 1,2-dimyristoyl-phosphatidylethanolamine-*N*-(polyethylene glycol-triethoxysilane (DPS) and the HIV-FP with the sequence AVGIGALFLGFLGAAGSTMGAASGGGKKKKK were custom synthesized by Shearwater Polymers (Huntsville, AL) and by the Yale WM Keck Biomolecular Research Facility (New Haven, CT), respectively.

### Preparation of GUVs

GUVs were prepared by the electroformation technique. In brief, 25 μl of a 10-mM lipid solution in organic solvent containing the fluorescent lipid probe Rh-PE (0.1 mol%) was deposited on clean glass slides that were coated with indium tin oxide and then placed in vacuum for 90 min to eliminate residual solvent. The fabrication chamber filled with 250 mM sucrose in H_2_O was composed by two conducting slides separated by a spacer of 0.5 mm. Electroformation was performed at around 60 °C by applying alternating electric current (3 V, 10 Hz) for 120 min. The GUVs were transferred into a 250-mM glucose solution to let them settle by gravity on the microscope slide.

### Preparation of supported membranes

Supported monolayers were prepared using the Langmuir–Blodgett technique. Quartz slides (Quartz Scientific, Fairport Harbor, OH) were cleaned by boiling in Contrad detergent for 15 min and then sonicated for 30 min in a hot bath. After rinsing with water and ethanol, remaining organic residues were removed by Piranha solution (3:1 of 95% H_2_SO_4_ 30% H_2_O_2_), followed by extensive rinsing in pure water. The quartz slides were further cleaned for 10 min in an argon plasma sterilizer (Harrick Scientific, Ossining, NY) immediately before use. Lipid mixtures dissolved in chloroform/methanol were deposited and spread onto the air–water interface of a Nima 611 Langmuir-Blogdett trough (Nima, Coventry, UK) at room temperature. The initial surface pressure was ∼5 mN m^−1^ and the lipid monolayer was equilibrated for 10 min to evaporate the solvent. After the monolayer was compressed at a velocity of 10 cm^2^ min^−1^ to reach a surface pressure of 32 mN m^−1^, the cleaned quartz slide was rapidly (200 mm min^−1^) dipped into the trough and slowly (5 mm min^−1^) withdrawn while keeping a constant surface pressure. The lipid monolayer transferred onto the quartz support was visualized by epifluorescence microscopy. SLBs were formed by a combined Langmuir–Blodgett (LB)/vesicle fusion technique^51^. Briefly, a lipid monolayer composed of the desired lipid composition and compressed to 32 mN m^−1^ was transferred from the air–water interface of a Langmuir trough onto a plasma-cleaned quartz slide. The LB monolayer contained 3% DPS to covalently link it to the SiO_2_ surface on the quartz slide by drying the coated slides in a desiccator at room temperature overnight. Slides with tethered polymer-supported LB monolayers were placed in a custom-built flow-through chamber. A 0.1-mM suspension of LUVs in HEPES buffer (10 mM HEPES, 120 mM NaCl, pH 7.2) were injected into the chamber and incubated for at least 2 h. Excess LUVs were washed out by extensive rinsing with HEPES buffer.

### Lipid and content mixing of LUVs

LUVs were prepared by extrusion through polycarbonate filters with 100 nm pores in HEPES buffer. The lipid mixing assay was based on a commonly used fluorescence resonance energy transfer assay^52^. LUVs were added to a cuvette in a ratio of 1:9 of labelled (1 mol% Rh-PE and N-(7-nitro-2-1,3-benzoxadiazol-4-yl)-dioleoyl-phosphatidylethanolamine (NBD-PE) each) to unlabelled LUVs to give a total lipid concentration of 50 μM in HEPES buffer at room temperature. Lipid mixing induced by HIV-FP was recorded under constant stirring using a Fluorolog-3 spectrofluorometer (Jobin-Yvon, Edison, NJ) with the excitation and emission wavelengths set at 460 and 535 nm, respectively. Fluorescence intensities of the LUV suspension alone and after the addition of Triton X-100 were defined as 0 and 100% lipid mixing, respectively. The content mixing of LUVs was evaluated as the decrease in fluorescence from ANTS due to quenching by DPX, as described previously^53^. LUVs containing either 25 mM ANTS or 90 mM DPX (10 mM HEPES, pH 7.2, 250 mmol kg^−1^ osmolality by adding of NaCl) were prepared and then unencapsulated materials were removed by using a PD-10 desalting column. Both LUVs were added to a cuvette at a 1:1 ratio to give a total lipid concentration of 400 μM in HEPES buffer at room temperature. The fluorescence intensity of ANTS at 520 nm with an excitation at 360 nm was measured under stirring after addition of HIV-FP. Vesicle contents mixing (0%) was defined as the fluorescence intensity of a 1:1 mixture of ANTS and DPX vesicles and 100% mixing of contents corresponded to the fluorescence intensity of a vesicle standard containing coencapsulated ANTS (12.5 mM) and DPX (45 mM).

### Membrane fusion of LUVs with SLB

To monitor membrane fusion of LUVs with SLBs, SLBs were incubated with 5 μM HIV-FP for 10 min and then unbound peptides were washed away with HEPES buffer. Different types of LUVs labelled with 0.5 mol% DiD were added to the SLB. The docking/fusion of LUVs to SLBs was monitored by TIRF microscopy^22^ and images were analysed using a homemade programme written in LabView (National Instruments). First, the whole stack of images was filtered by a moving average filter. The intensity maximum for each pixel over the whole stack was projected on a single image. LUVs were located in this image by a single-particle detection algorithm^54^. This entailed that trajectories of individual particles were reconstructed by comparing the results of successive images of each series. Only trajectories with at least four time steps (five data points) were used in the presented analysis. Although this reduced the number of available traces, it improved the analysis and avoided artifacts from noise and photobleaching. The peak (central pixel) and mean fluorescence intensities of a 5 × 5 pixel^2^ area around each identified center of mass were plotted as a function of time for all particles in images of each series. The exact time points of docking and fusion were determined from the central pixel^55^.

## Additional information

**How to cite this article**: Yang, S.-T. *et al*. Line tension at lipid phase boundaries as driving force for HIV fusion peptide-mediated fusion. *Nat. Commun.* 7:11401 doi: 10.1038/ncomms11401 (2016).

## Supplementary Material

Supplementary Figures Supplementary Figures 1-5

Supplementary Movie 1 Binding of HIV-FP-decorated LUVs to Lo/Ld domain boundaries on a GUV observed by epifluorescence microscopy. LUVs composed of brain PC/brain PS (bPC/bPS, 3:1) were preincubated with HIV-FP and added to the phase-separated GUV consisting of DPPC/DOPC/DOPS/Ch (2:1:1:1). The bound liposomes are seen to move along with the Lo/Ld interfaces on the GUV. Total time is 20 sec and each frame is 35 x 35 μm^2^.

Supplementary Movie 2 Membrane fusion of LUVs with a SLB observed by TIRF microscopy. Four kinds of LUVs and SLBs were composed of saturated lipid/DOPC/DOPS/Ch (2:1:1:1). DLPC, DMPC, DPPC, and DSPC are used as saturated lipids. LUVs were labeled with 0.5 mol% DiD to monitor single vesicle fusion events with SLBs. The fraction of fusion events (of total docking plus fusion events) increases with acyl chain lengths in the order DLPC < DMPC < DPPC < DSPC, indicating that line tension plays a significant role in the fusion process. Total time is 519 sec and each frame is 4 times (55 x 55) μm^2^ from left to right: DLPC, DMPC, DPPC, and DSPC.
