# Supplementary material for: Line tension at lipid phase boundaries as driving force for HIV fusion peptide-mediated fusion
Source: Nat Commun. 2016 Apr 26;7:11401. doi: 10.1038/ncomms11401 (PMC4853434; doi:10.1038/ncomms11401)
Supplement: Supplementary Figures — 1-5 [file ncomms11401-s1.pdf]

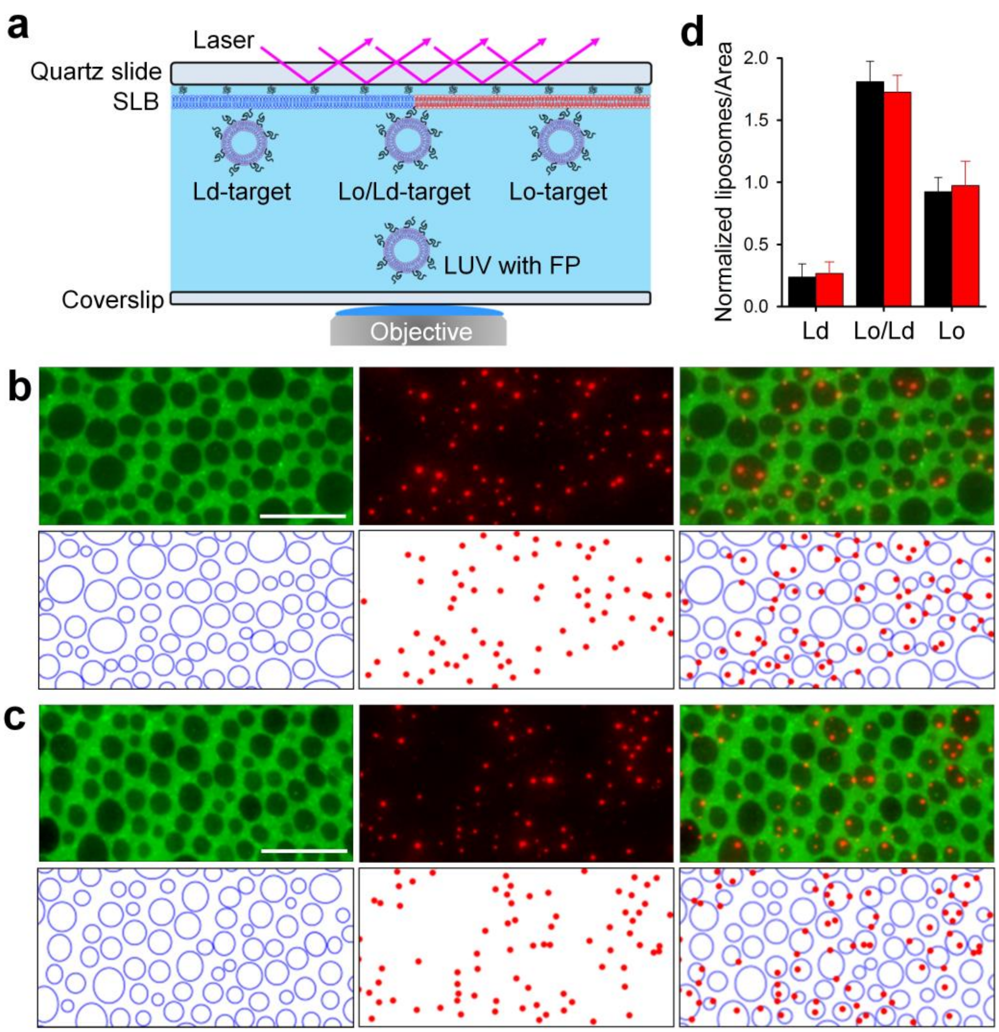

Supplementary Figure 1. Targeting of HIV-FP-decorated LUVs to coexisting Lo/Ld phase domains in SLBs. (a) Schematic diagram showing the TIRF microscopy setup to measure the targeting of HIV-FP-liposomes to SLBs. LUVs were pre-incubated with HIV-FP and then allowed to bind to SLBs. (b and c) Epifluorescence micrographs (top left) of SLBs composed of (b) PSM/DOPC/Ch (2:1:1) or (c) DPPC/DOPC/Ch (2:1:1) labeled with 0.1 mol% Rh-PE and TIRF micrograph (top center) of bound liposomes composed of brain PC/brain PS (bPC/bPS, 3:1) labeled with 0.5 mol% DiD. The overlay image (top right) shows that most liposomes favor the boundaries between Lo and Ld phases. Circular boundaries around each Lo domain are defined (bottom left) and the x/y coordinates of each membrane-bound liposome are determined by fitting a 2D Gaussian curve to the point spread function (bottom center) and overlayed (bottom right). Scale bars are 20 μm. (d) Distribution of bound liposomes to three different regions (Ld, Lo, and a 0.75 μm wide boundary region) of SLBs. Data are mean ± s.d. from three experiments.

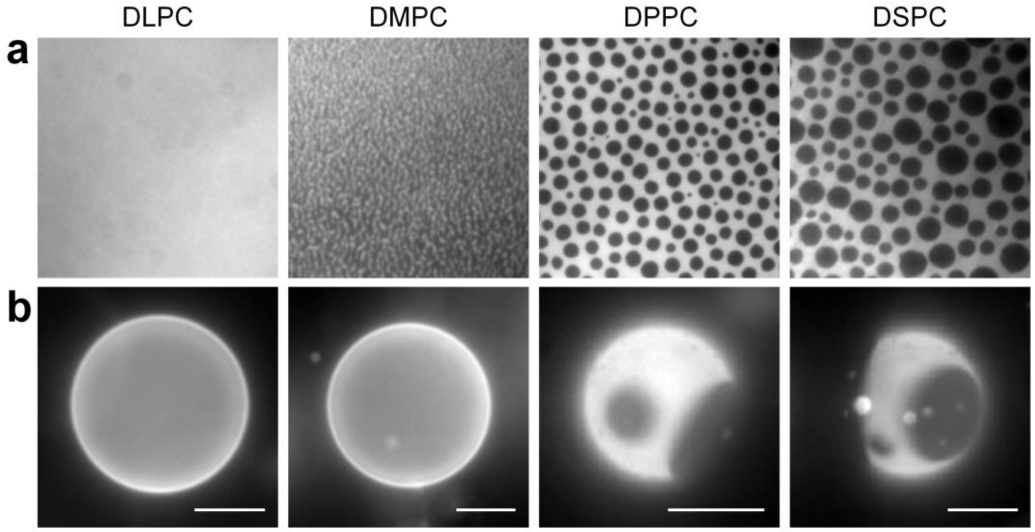

Supplementary Figure 2. Effect of hydrophobic mismatch on lipid phase behavior. Fluorescence micrographs of (a) lipid monolayers and (b) GUVs composed of saturated PC/DOPC/DOPS/Ch (2:1:1:1) labeled with 0.1 mol% Rh-PE. Saturated PCs with different acyl chain lengths are indicated. The scale of all monolayers is 60 x 60 μm^2 and scale bars in GUV images are 10 μm.

a

	Numbers of Lo	Total area of Lo (μm^2)	Area of Lo (μm^2)	Radius of Lo (μm)	Circumference of Lo (μm)	Lo fraction (%)
Control	153 ± 15	1632 ± 190	10.6 ± 0.7	1.84 ± 0.06	11.5 ± 0.4	39.8 ± 4.6
POPC	169 ± 12	1474 ± 103	8.7 ± 0.5	1.66 ± 0.05	10.4 ± 0.3	35.9 ± 2.5
POPE	127 ± 14	1629 ± 70	12.8 ± 1.9	2.01 ± 0.14	12.7 ± 0.9	39.7 ± 1.7
POPG	169 ± 8	1421 ± 71	8.4 ± 0.8	1.64 ± 0.07	10.2 ± 0.5	34.7 ± 1.7
POPS	224 ± 17	1098 ± 59	4.9 ± 0.2	1.25 ± 0.03	7.9 ± 0.2	26.8 ± 1.4
α-TOH	87 ± 14	250 ± 25	2.9 ± 0.4	0.96 ± 0.06	6.0 ± 0.4	6.1 ± 0.6

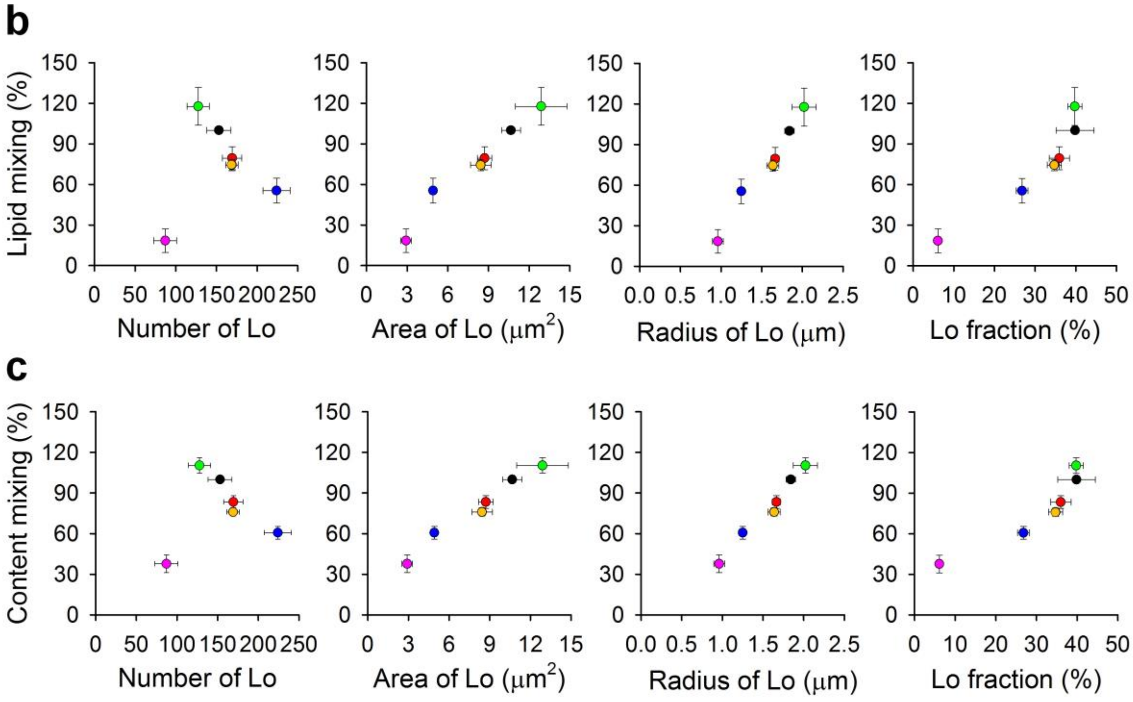

Supplementary Figure 3. Relationship between membrane fusion and various properties of linactant-modified Lo domains. (a) Effect of hybrid phospholipids and α-TOH on Lo domains in supported DPPC/DOPC/DOPS/Ch (2:1:1:1) monolayers. Several parameters of Lo domains based on multiple representative images such as those shown in Figure 6a were analyzed by ImageJ. The values are mean ± s.e.m. from three experiments. Correlations of (b) lipid or (c) content mixing with number, area, radius, and fraction of Lo domains. Relative extents of lipid and content mixing are shown in Figure 6b. The colored data points in b and c correspond to the colors of the added linactants in a.

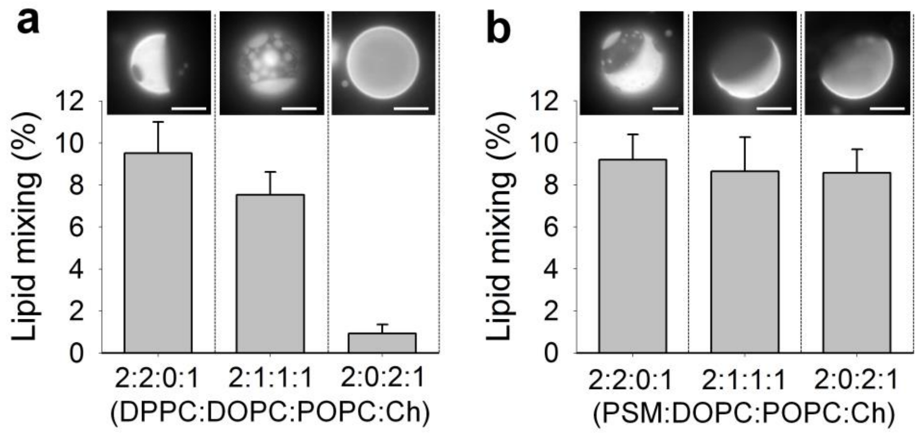

Supplementary Figure 4. Effect of POPC on lipid phase separation and membrane fusion in uncharged membranes. Lipid mixing induced by HIV-FP and epifluorescence micrographs of GUVs composed of (a) DPPC/DOPC/POPC/Ch and (b) PSM/DOPC/POPC/Ch with indicated ratios. Scale bars are 10 μm. The extent of lipid mixing was measured 30 min after addition of 5 μM HIV-FP to 100 μM LUVs. Data are mean ± s.d. from three experiments.

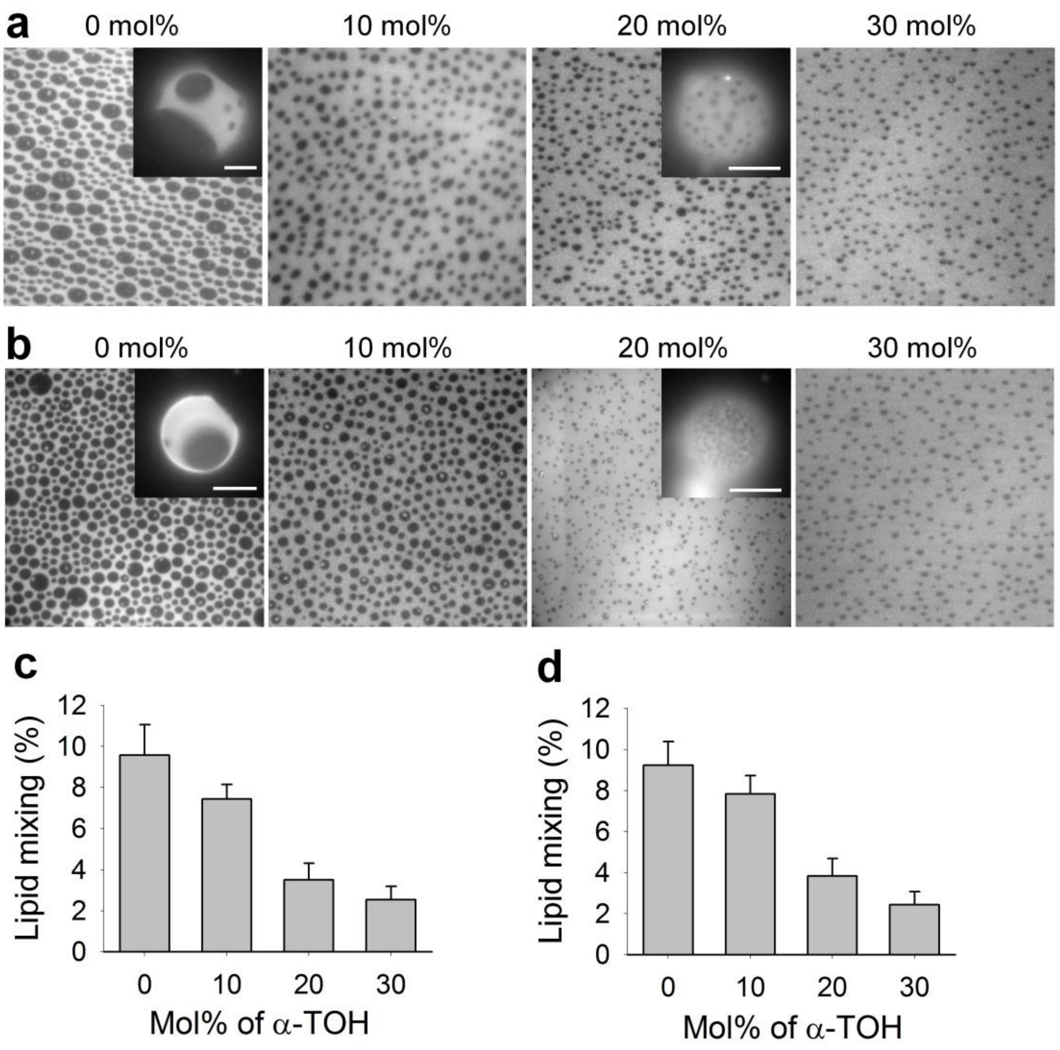

Supplementary Figure 5. Effect of α-TOH on lipid phase separation and membrane fusion in uncharged membranes. Epifluorescence micrographs of lipid monolayers and GUVs composed of (a) DPPC/DOPC/Ch (2:2:1) and (b) PSM/DOPC/Ch (2:2:1) containing the indicated mol% α-TOH. The scale of all monolayers is 64 x 64 μm^2 and scale bars in the GUV images are 10 μm. The domain sizes decrease with increasing mol% α-TOH. (c and d) Effect of α-TOH on lipid mixing mediated by HIV-FP. The extent of lipid mixing was measured 30 min after addition of 5 μM HIV-FP to 100 μM LUVs composed of (c) DPPC/DOPC/Ch (2:2:1) or (d) PSM/DOPC/Ch (2:2:1) with indicated mol% α-TOH. Data are mean ± s.d. from three experiments.
